# Effects of Comorbid Disease Improvement on Oral Lichen Planus (OLP) and Oral Leukoplakia (OL) Lesions: A Retrospective Longitudinal Study

**DOI:** 10.3390/jcm14103408

**Published:** 2025-05-13

**Authors:** Ildikó Tar, Szarka Krisztina, Renáta Martos, Csongor Kiss, Ildikó Márton

**Affiliations:** 1 Department of Oral Medicine, Faculty of Dentistry, University of Debrecen, Nagyerdei krt. 98, 4032 Debrecen, Hungary; 2 Institute of Metagenomics, University of Debrecen, Nagyerdei krt. 98, 4032 Debrecen, Hungary; szkrisz@med.unideb.hu; 3 One Health Institute, Faculty of Health, University of Debrecen, Nagyerdei krt. 98, 4032 Debrecen, Hungary; 4 Department of Operative Dentistry and Endodontics, Faculty of Dentistry, University of Debrecen, Nagyerdei krt. 98, 4032 Debrecen, Hungary; martos.renata@dental.unideb.hu; 5 Division of Pediatric Hematology-Oncology, Department of Pediatrics, Faculty of Medicine, University of Debrecen, Nagyerdei krt. 98, 4032 Debrecen, Hungary; kisscs@med.unideb.hu; 6 Department of Biochemistry and Molecular Biology, Faculty of Medicine, University of Debrecen, Nagyerdei krt. 98, 4032 Debrecen, Hungary; marton.ildiko@dental.unideb.hu; 7 Scientific Advisor of the Faculty of Health, University of Debrecen, Nagyerdei krt. 98, 4032 Debrecen, Hungary

**Keywords:** oral lichen planus, oral leukoplakia, HPV, comorbid disease, prevention of cancer formation

## Abstract

**Background:** Previous attempts to treat oral potentially malignant disorders OPMDs) effectively have failed. Longitudinal studies investigating the effects of comorbid diseases improvement on OPMDs are not yet available. Therefore, the current study examined the effects of comorbid disease improvement on OPMDs healing, both in oral lichen planus (OLP) and oral leukoplakia (OL) patients. **Methods:** The data from 197 consecutive patients (144 females and 53 males, age ± SD: 55.19 ± 12.37 years, with ages ranging from 23 to 91 years), with oral lesions considered OLP and OL, were processed and evaluated. The frequency of comorbid diseases and the presence of HPV (here, subtypes were not evaluated) in the lesions in OLP and OL patient groups were evaluated and compared to the results of controls (*n* = 139). Risk models for OLP and OL lesions were established. High-risk models for erosive–atrophic OLP and non-homogeneous OLP were also described. The influence of comorbid disease improvement was also evaluated. Lesions were scored at the first and last visit (full recovery = 0, improvement = 1, and no improvement = 2). **Results:** One hundred and ninety-seven patients (144 OLP + 53 OL) were followed up for an average of 47.66 months (min–max: 1–203 months, SD: 54.19). Based on the established models, HPV infection, iron deficiency, diabetes, and thyroid function disorders seem to act as risk factors for OLP and may also affect OL formation. The improvement in comorbid diseases can cause significant improvement in OLP and OL lesions. **Conclusions**: By meticulous follow-up of comorbid diseases, improvement in OLP and OL lesions can be achieved.

## 1. Introduction

Oral cancer development is a worldwide healthcare problem [1]. The appearance of oral precancerous lesions, or, as more commonly preferred nowadays, oral potentially malignant disorders (OPMDs), precedes the development of oral cancers. One-third of developed oral cancers originate from previously diagnosed and followed-up oral precancerous lesions [2].

The OPMD most often leading to cancer formation are oral lichen planus (OLP) and oral leukoplakia (OL). In the case of OLP, tumour progression occurred in 0.44–2.29% of cases [3,4]. In the case of OL, a meta-analysis had shown about 4.47–10.74% of OL cases progressing to oral cancer. The highest malignancy rates of OPMDs were shown in Asia. Being atrophic and/or erosive in the long term, the lesion had a higher probability of progressing to malignancy. Thereby, non-homogeneous OL and erosive OLP have a higher probability of turning malignant. Additional risk factors were smoking, drinking alcohol, being a male, and in some studies, having HCV infection also increased the malignant potential [3,5].

Nowadays, OPMD treatment can be described as the elimination of all recognised local factors, alongside the application of topically administered drugs or excision of the lesion if it small. Afterwards, in routine medical practice, the thorough follow-up of lesions is conducted to intervene in their progression as early as possible [6]. As mentioned before, the presence of atrophy and erosion, which means that an increased rate of cell death in the lesion, with a higher probability of mistakes in cell division, also increases the probability of cancerisation.

The main approach, as well as the challenge of OPMD therapy, as in most therapies, is the cooperation of the affected patient. If they do not want to quit smoking or drinking or do not intend to keep good oral hygiene, it is hard to eliminate or improve the condition of OPMDs. Earlier, in the case of OLP, it was stated that local topical treatments were also among the less effective therapies; practically, they were not superior to the placebo [7,8]. Currently, the situation has not improved at all. Although biological therapies have emerged in the everyday practice in multiple specialities, their side effects and their safety are still not carefully examined, especially in the case of OPMD [8].

For OLP, some drugs (such as steroids, calcineurin inhibitors, retinoids, and anti-tumour necrosis factor (TNF) α therapies) must be used with serious precautions for topical, oral, or intravenous application, as they have deteriorating effects on certain comorbid diseases such as hypertension and diabetes, or, in some cases, they are fully contraindicated (breast feeding). In the case of tacrolimus and pimecrolimus, which are calcineurin inhibitors, long-term use may also initiate cancer formation, and they are not exceptional amongst biological therapies with this type of potential. At the very least, they can be used as acute solutions to reduce the length of severe outbreaks and alleviate related pain [9]. The other controversy that exists with anti-TNFα biological therapies (which might also be a treatment option in OLP cases) is that they may cause OLP formation as a result of reducing TNFα levels, with the parallel increase in interferon α (IFN) levels, which can create an environment that is favoured by T cell proliferation, leading to OLP eruptions [10]. The same phenomenon occurs in patients with infectious hepatitis (HBV, HCV) who receive antiviral therapy with ribavirin and IFN α, creating an environment favoured by T lymphocytes. OLP eruptions are also common, even in this case.

In the case of OL, even the complete elimination of all known etiological factors can lead to partial healing in quantity and quality [11]. In the 1960s and 1970s, the role of human papillomavirus (HPV) in cancer formation was still in the shadows. Still, alcohol consumption, bad oral hygiene, and smoking habits were already among the known risk factors. Hypothetically, proper treatment, which involves all previously known etiological factors could have eliminated these lesions. Reviews on OL treatment options state that the active treatment with vitamin A, bleomycin, β carotene, and lycopene does not reduce the possibility of cancer formation more than a placebo, although they sometimes do provide at least temporary improvement [12,13].

Regarding OLP, the relationship with comorbid diseases is already known [14]. For OL, the effects of comorbid diseases are not that obvious, though the connection is more than probable, and the interaction between etiological factors of OL and systemic diseases cannot be ruled out [15]. Longitudinal studies that investigate the effects of comorbid disease improvement on OPMDs are not yet available. Therefore, the aim of the current study is to examine the effects of comorbid disease improvement on the healing of OPMDs both in OLP and OL patients.

Hypothesis: Improvement in comorbid diseases is expected to enhance the healing of OLP and OL lesions in patients.

Steps to take:

1. To determine the frequency of comorbid diseases in patients with OLP and OL and to compare it to that in patients without oral mucosa lesions. Is the frequency of comorbid diseases higher in any of the groups (OLP, OL, and control)?
2. To decide whether certain diseases are more frequent in OLP with erosive atrophic subtypes or in patients with non-homogeneous OL.
3. To establish a risk model for OLP and OL lesions, a high-risk model for erosive atrophic OLP and non-homogeneous OL, and a risk model for specific locations (gingiva and lip involvement) of the previously mentioned disorders.
4. To examine whether comorbid disease improvement affected the improvement in OLP or OL lesions and to identify the outcomes.

## 2. Materials and Methods

### 2.1. Patients and Investigations

Between 1 January 1996 and 31 December 2016, the data of 197 consecutive patients (144 females and 53 males, age ± SD: 55.19 ± 12.37 years, ranging between 23 and 91 years), with oral lesions diagnosed as OLP and OL, attending at the Department of Periodontology, Faculty of Dentistry, Medical and Health Science Centre, University of Debrecen, were processed and evaluated in this study. Investigations were performed after having obtained written informed consent from the patients. This study was approved by the local clinical ethical committee of the university (Ethics Cometee-2247-2004, Debrecen, 8 November 2004).

The diagnosis of OLP was made based on clinical diagnostic criteria, including more or less symmetrical lesions and the presence of Wickham’s striae. The lesions can be categorised as reticular, popular, plaque-like (non-erosive, non-atrophic types), or atrophic, erosive, or bullous types (atrophic–erosive types). Histological findings included the presence of ribbon-like subepithelial lymphocyte infiltration, liquefaction degeneration (exocytosis), and hyperkeratosis with the thickening of the granular cell layer.

The diagnosis of OL was made based on clinical appearance as a non-wipeable, predominantly white plaque that cannot be attributed to any other disease. At the first visit, the elimination of mechanical factors that can cause such lesions and blood testing for the possible systemic causes were performed. Two weeks after the elimination of mechanical irritation factors such as ill-fitting dentures, sharp edges of teeth, a clinical check-up was conducted. If there was no change, a biopsy was performed. Smaller lesions (2 cm ≥ in diameter) with easier access for excisional biopsy were removed. These patients were not included in this study. Only patients with bigger size lesions and incisional biopsy were included in this study. The minimum time needed for this process was one month. The OL lesions were categorised as homogenous and non-homogenous OL that included speckled, nodular and verrucous types. All of them contain more or less erythematous areas with atrophy and/or erosion (erythroplakia). Non-homogeneous forms generally have a higher risk of malignant transformation compared to homogenous forms.

Though data in this study were collected retrospectively, the clinical description of the lesions [15,16,17], including types and location on oral mucosa, histological results with detailed descriptions, patients’ history, results of repeated blood tests, HPV cytology [18], and patch test results for dental materials, was also available for these patients (Table 1). HPV, EBV, and TTV detailed data of this patient group, cytology, and examination methods were published earlier [18,19,20,21,22]. Current detailed data of virus prevalence are not shown in this study. Only the presence of the condition in the lesion, excluding patients with healthy mucosa (+/−), was used as data point for model establishment. Testing for anti-HbsAg, anti-HbcAg, anti-HbeAg, and anti-HCV was also conducted, except in cases of previously diagnosed disease. Previous tumours (patient history), and tumours found throughout the course of this study were also registered.

Patient care involved symptomatic local therapy of the oral lesions provided by the dentist participating in this study and lege artis treatment (according to guidelines) of the coinciding systemic diseases, performed by the general practitioners of the patients or by competent specialists. Topical antiseptic treatment, analgesics, and gels that physically protect eroded surfaces were applied to prevent bacterial and/or fungal superinfection of erosive OLP lesions or non-homogeneous OL. The use of local corticosteroids, vitamin A oil, calcineurin inhibitors, other preparations, and systemic therapies intended to treat the oral lesions, was avoided. Patients were advised to return for follow-up exams every three months. Deterioration and recurrence of the lesions, as well as the emergence of any novel complaint, were indications for an immediate check-up. All these were described in the patients’ documents. The presence of non-plaque-induced gingival inflammation, named desquamative gingivitis, and lip involvement was also registered.

Repeated blood testing was also part of the OPMDs checkup (Table 1), or blood test results within 1 month were accepted from patients, and their results were also added to the patients’ files. Patients with deficiency conditions (iron, folic acid, B12) were instructed to take iron or vitamin supplements. While patients with glucose intolerance (120’OGTT result over 7.8 mmol/L + HbA1c under 7%) were instructed regarding their diet.

Other treatments were introduced by specialists because of using prescribed drugs, many of which can be prescribed by specialists, and in such cases patients receive a discount under local health insurance regulations. During check-up examinations (every three months), the patients’ notes from other specialists were also requested and added to the dental check-up notes. Specialists were responsible for applying their own disease-specific guidelines, including the criteria for diagnosis and for assessing patient improvement.

Improvement in the comorbid disease was evaluated by the specialist who followed up with the patient and prescribed their medication. Improvement was scored by comparing repeated test values in terms of systemic diseases or oral lesions (full recovery = 0, improvement = 1, and no improvement = 2). The presence of lesions was scored as follows: no clinical lesion = 0; non-erosive–atrophic OLP or homogenous OL = 1; and erosive–atrophic OLP or non-homogenous OL = 2, as in a previous study [19].

### 2.2. Control Subjects

One hundred and thirty-nine subjects (96 females and 43 males; age ± SD: 52.52 ± 14.06; min-max: 16–82) with intact oral mucosa and no oral complaints were enrolled in this study. The history of previous and current diseases, together with blood test results, was registered.

### 2.3. Statistics

Patients were classified according to oral diagnosis. For statistical evaluation, binary data were collected, except for age and follow-up time. Prevalence of comorbid diseases and the presence of HPV were evaluated [18,19,20,21,22]. Between-group differences were also tested (ANOVA, Tukey’s post hoc test) (hypothesis questions 1–2). Possible associations between the presence of OLP and OL lesions and coinciding systemic diseases (any risk and high-risk models), anatomical localisation of lesions (gingiva and lip), and HPV infection were assessed by binary logistic regression analysis (hypothesis question 3). A comparison of treatment results was made with paired-sample *t*-tests. The relationship between the improvement scores for systemic diseases and the improvement scores for lesions was examined using Spearman’s rank correlation (hypothesis question 4). SPSS version 20.0 (SPSS Inc., Chicago, IL, USA) was used for statistical analysis.

## 3. Results

### 3.1. Group Characteristics and Comorbid Disease Prevalence

One hundred and forty-four OLP patients (mean age ± SD: 54.56 ± 12.37 years; min–max: 23–80 years; 109 females and 35 males) and 53 OL patients (mean age ± SD: 56.91 ± 12.31 years; min–max: 29–91 years; 35 females and 18 males) were enrolled in this study. The control group had a mean age ± SD: 52.52 ± 14.06 years; min–max: 16–82 yrs; 96 females and 43 males. The OLP, OL, and control groups did not differ significantly in terms of age (ANOVA, *p* = 0.11; Tukey, *p* = 0.08) or gender (ANOVA, *p* = 0.23; Tukey, *p* = 0.28). Ninety-three (64.5%) patients had the non-erosive, non-atrophic form, while 51 (35.5%) patients had an atrophic–erosive form of OLP. Thirty-six (67.9%) OL patients had homogenous, whilst 17 OL patients (22.1%) had a non-homogenous form of OL (Table 2).

As seen in the frequency table with percentiles, the most common diseases found in OLP, OL, and control groups were cardiovascular diseases, including hypertension, fibrillation, and valve problems, such as CVD. Eighty OLP patients (55%), 26 (49%) OL patients, and 57 (41.7%) control subjects had CVD. The prevalence of CVD in both disease groups was statistically the same.

The frequency of other diseases (Table 2) or conditions that also did not show a significant difference (*p* > 0.05) between groups were smoking (*p* = 0.79), anaemia (*p* = 0.29), vitamin B deficiency (*p* = 0.1), hyperlipidaemia (*p* = 0.09), thyroiditis (Hashimoto and Graves) (*p* = 0.55), the frequency of systemic autoimmune diseases (including Sjögren’s syndrome, Raynaud’s syndrome, rheumatoid arthritis, mixed connective tissue disease) (*p* = 0.08), and allergy to dental materials (nickel, thiomersal, phenylmercuribete, and mercury allergies occurred) (*p* = 0.76).

Significant differences (*p* < 0.05) existed between the control group and patients’ groups in the case of prevalence of HPV (*p* = 0.00) and iron deficiency (*p* = 0.00). In case of OLP patients, the frequency of diabetes (both type I and II) (*p* = 0.01), and thyroid function disorders (hypo- and hyperfunction) (*p* = 0.05) were significantly higher among OLP patients than in OL patients and controls. In OL patients, the only significant difference compared to the other two groups was observed in liver function disorders involving mostly toxic hepatitis cases and a few cases of infectious hepatitis: there were two cases of chronic active hepatitis (HCVRNA positive) among the three anti-HCV positive patients (*p* = 0.02) (Table 2).

There was no significant difference in the prevalence of previously found tumours (*p* = 0.16), and, there was no difference between groups according to the post hoc test. In the OLP patients, previous findings included four low- and high-grade cervical epithelial lesions, one mammary, one melanoma, one colon tumour, and one OSCC of the buccal mucosa, while in the OL patients, one meningeal and one endometrial tumour were revealed earlier. During the course of the current diagnostic procedure, one kidney and one prostate tumour were found. By their removal, the OLP lesion regressed to a non-erosive–non-atrophic state.

In both patient groups, desquamative gingivitis or gingival location and lip involvement were also present. In the OLP group, 25/144 (17.3%) patients had desquamative gingivitis, and 8/144 (5.5%) patients had lip involvement. In the OL group, 8/53 (20.7%) patients had gingival involvement, and 2/53 (3.7%) had lip involvement. There was no significant difference between the OLP and OL groups in terms of the prevalence of gingival and lip involvement (Table 2).

### 3.2. Prevalence of Comorbid Diseases in OLP Subtypes (Non-Erosive–Non Atrophic and Erosive–Atrophic Types), and in OL Subtypes (Homogenous and Non-Homogenous Subtypes)

Comparison of comorbid disease prevalence in OLP and OL subtypes revealed that only diabetes prevalence was significantly different (*p* = 0.004) between OLP subtypes. All other diseases showed no significant differences between subtypes (Table 3).

### 3.3. Predisposing Factors for OLP and OL (Any Risk Model)

Regression analysis involving data from OLP and control patients revealed that some of the examined systemic factors may work as a predisposing factor for OLP. HPV infection (*p* = 0.00; Exp(B): 8.61), iron deficiency (*p* = 0.00; Exp(B): 6.07), diabetes (*p* = 0.01; Exp(B): 5.2), and thyroid function disorders (*p* = 0.01; Exp(B): 2.81) seem to function as risk factors for OLP.

A bit different composition of systemic predisposing factors (involving OL and control patients’ data) was found in case of OL: HPV infection (*p* = 0.00; Exp(B): 12.29), iron deficiency (*p* = 0.00, Exp(B): 5.53), and diabetes (*p* = 0.013, Exp(B): 5.07).

### 3.4. Possible Predisposing Factors for Atrophic–Erosive OLP or Non-Homogeneous OL (High-Risk Model)

The main risk factor (all OLP subtypes data) for erosive forms of OLP is diabetes (*p* = 0.026; Exp(B): 2.93). In the case of non-homogeneous OL, no specific systemic risk factor was identified from the examination.

### 3.5. Lip Involvement

In OLP patients, lip involvement seems to be affected by gender (*p* = 0.014; Exp(B): 9.17), by smoking (*p* = 0.003; Exp(B): 0.023), and by diabetes (*p* = 0.011; Exp(B): 9.89). In OL patients, no systemic predisposing factor could have been detected among the examined factors.

### 3.6. Desquamative Gingivitis

In OLP, thyroiditis provides the highest impact on desquamative gingivitis (*p* = 0.00; Exp(B): 19.67). In OL, there is no specific systemic factor amongst the investigated factors that may influence this type of location.

### 3.7. Improvement in Co-Morbid Diseases and Lesions in OLP and OL Patients

One hundred and ninety-seven patients (144 OLP +53 OL) were followed up for an average of 47.66 months (min–max: 1–203 months, SD: 54.19). At the beginning of the study period, in the OLP group, there were 91 patients with non-atrophic–non-erosive lesions, while 53 had the atrophic–erosive form. Amongst the OL patients enrolled in this study, 38 patients were classified as having homogenous OL, while 15 patients started the study period with non-homogenous OLs.

By the end of this study, regarding systemic conditions across all 197 patients (144 OLP and 53 OL), 11 had not shown any improvement, 82 had shown partial improvement, and 104 had full recovery. Seventy-nine OLP patients fully recovered from comorbid conditions, 59 partially improved, and six did not improve at all. In the case of OL patients, 25 showed full recovery, 23 had mild improvement, and the rest (n = 5) did not improve at all from the systemic condition.

In parallel, the lesions also regressed to a better condition: in the case of OLP, the lesion clinically disappeared in 38 patients, while in 106 patients, the lesion was in a non-erosive–non-atrophic form (Table 4). In five patients with OLP, there was no change, but these patients had non-erosive–non-atrophic forms even at the beginning of this study. In one female OLP patient, the lesion fluctuated between non-erosive–non-atrophic and erosive–atrophic forms. She had Hashimoto’s thyroiditis with hypofunction, never smoked, and she also abstained from alcohol consumption. After nearly 17 years of follow-up (in which her compliance was not fully satisfactory), there was a lymph node enlargement in the submandibular area, on the right side. The biopsy from the OLP lesion did not even show any dysplasia; it just showed the histological characteristics of OLP, but the lymph node biopsy confirmed OSCC origin. She was referred to maxillofacial oncology.

There was another male patient who quit follow-up after three years (at that point having the non-atrophic–non-erosive OLP form present in small areas along the tongue margin). Then, after five years, he was back with an erosive–atrophic lesion on the right side of the tongue. Biopsy confirmed an in situ carcinoma. He was also referred to maxillofacial oncology. After the tongue surgery, the OLP disappeared from the oral cavity. After two years, OLP recurred, so he has been under dual monitoring ever since.

In the case of OL, 18 lesions disappeared (13 homogenous and five non-homogenous OL at the beginning of this study). Thirty-five OL lesions presented in a homogenous form at the end of the study period (Table 5). A comparison of lesion scores in OLP and OL patients at the beginning (mean ± SD: 1.34 ± 0.47) and at the end of the follow-up period (mean ± SD: 0.72 ± 0.46) showed a significant difference (*p* = 0.000). Improvement in lesions (OLP and OL) was significantly correlated with improvement in systemic diseases: R = 0.34, *p* = 0.000.

## 4. Discussion

In OLP and OL patients, the frequency of CVD seemed higher than in the controls, but the difference was not statistically significant [23]. Previous studies examined this relationship, though medication taken for diseases such as hypertension may have interfered with OLP; in the case of OL, the common risk factor could be smoking [24,25]. Strangely, there was no difference in the frequency of smokers, patients with folic acid and vitamin B12 deficiency, or those diagnosed with thyroiditis and autoimmune disease in each group. Earlier studies have not examined the prevalence of these conditions in comparison to subjects having healthy-appearing oral mucosa.

Both OLP and OL patients harboured significantly higher levels of HPV, and a significantly higher number of patients exhibited iron deficiency compared to controls. The presence of HPV in more lesions than in normal oral mucosa has been demonstrated in other studies, with the highest frequency observed in OL [18].

In OLP patients, diabetes and thyroid function disorders seem to occur with a higher prevalence, which emphasizes the importance of metabolic problems in OLP aetiology [25]. Both in diabetes types and OLP, the presence of autoreactive cells has been proven. Both CD4+ and CD8+ cells play a role in both aetiologies. In OLP, in the histological picture, mainly in the non-erosive–non-atrophic variants, most T cells express CD8+. In a study, it was shown that in erosive–atrophic variants, histology reveals the emergence of CD4+ cell expression alongside CD8+ cells, mainly perivascularly [26]. The other possible connection between OLP and diabetes is that the main metabolic route of these CD4+ and CD8 + lymphocytes is through glycolysis and the pentose phosphate pathways. If sugar take-up of lymphocytes, mainly T or B types, is disrupted, as seen in diabetes, their functions are not performed in the original way.

Though OL is primarily associated with local risk factors, liver function problems increase the co-occurrence of infections, and they also deteriorate iron metabolism and, consequently, haemoglobin production. Liver diseases also increase the probability of secondary diabetes and thyroid problems [27]. Liver function problems, mainly toxic ones, may also have a common aetiological factor with leukoplakia, namely alcohol [28].

HPV infection, iron deficiency, and diabetes seem to serve as risk factors for both OLP and OL formation. Thyroid function problems, as they could also be related to diabetes, provide additional risks for OLP formation, but not for OL. The major risk factor for erosive OLP is diabetes. This is in accordance with previous studies [25].

Iron deficiency affects the functions of T regulatory (Treg) cells and natural killer (NK) cells, while diabetes more strongly affects the function of CD8+ and CD4+ cells (Th1 and Th2). Iron also takes part in the regeneration and proliferation of epithelial cells by taking part in haemoglobin formation, oxygen transport, DNA synthesis, and mitochondrial respiration. It is also an important element in oxidative stress and the elimination of inflammation. In this study, in the case of OL, no certain factor among those examined was identified as having a significant role in the erosiveness of the lesion. It is likely that in this pool of patients with HPV infections, there were no predisposing genes that helped HPV multiplication. For the GCC haplotype of the interleukin-10 (IL-10) gene promoter, the clearance of high-risk HPVs was reduced. Lower production of IL-10 may impair the production of anti-inflammatory cytokines needed for the proper elimination of HPVs by Th1-Th2 immunoregulation. Certain HLA-DR types can also be tied to acquiring or eliminating HPVs [29].

Other studies have not tried to investigate the aetiology of lip involvement in OLP, although the frequency of lip involvement is the same across both OLP and OL groups. In OLP, the main influencing factors are gender, smoking, and diabetes. While gender and diabetes increase the likelihood, smoking seems to reduce the frequency. All of them may alter the immune function of patients [30]. Smoking and diabetes can affect regenerative capacity by reducing oxygen supply and changing immune functions. Smoking seems to suppress all types of immunoreactions, while diabetes mostly alters T and B cell activities, as mentioned before. Diabetes can also increase collagenase activity in tissues [31].

In the case of OL, none of the examined characteristics could be tied to OL lip involvement. In OL, lip involvement is mainly attributed to sun exposure rather than systemic causes, but smoking may have an augmenting effect [32].

Desquamative gingivitis seems to be a sign of thyroiditis in OLP patients. In Hashimoto thyroiditis, the serum levels of IL-2, IL-18, and IFNγ are higher in these patients. This is also true for OLP patients [33]. In Hashimoto’s, the immune response seems to be characterised by a Th1 pattern of cellular immunity. Certain gene polymorphisms serve as risk factors for OLP formation [33]. These are γ-interferon and TNFα genes, both of which take part in the activation of the Th1 cell pathway.

In the case of OL, none of the examined factors affect the gingival location [34]. In OL, it is more probable that the gingival location is more adherent to plaque-induced periodontal disease (gingivitis or periodontitis). Previous studies of OLP examined the prevalence of different thyroid diseases, primarily hypothyroidism and autoantibodies produced in thyroiditis [35].

We could relate the improvement in OLP and OL lesions to comorbid diseases. In general, the improvement in comorbid diseases did lead to improvement in the OL condition, but no specific diseases that had a major effect on OL lesions were identified. The pathogenesis of OL lies more in local mucosal irritation, with chronic inflammation occurring secondary to local irritation.

At first sight, the role of comorbid diseases in healing does not seem to be important, but their effect on regeneration cannot be denied. Furthermore, iron and vitamin B deficiencies and overload are considered to be precancerous conditions that may promote tumour genesis [35,36,37]. Diabetes also poses a risk for tumour formation due to reduced regenerative capacity and altered immunological reactions [38]. However, as with many conditions, the treatment of diabetes largely depends on patient cooperation. Patients must be motivated enough to take the prescribed medications at the given times. Lifestyle and habit changes are also major contributors of the treatment process. Diabetic patients are advised to change their diet to control sugar intake and exercise regularly.

The aforementioned and evaluated therapy for OLP and OL lesions targets systemic diseases, all of which, by elevating inflammatory cytokines or causing deficiencies in basic nutritional elements such as vitamins or macro- and microelements, reduce the regenerative and protective potential of the oral mucosa [37,39]. Hyperkeratosis, atrophy, and erosion are the basic histological phenomena of both OLP and OL lesions, showing an increasing apoptotic rate, respectively [40,41]. Systemic diseases also have the same effect on the lesions by augmenting the increase in their apoptotic rate. Conclusively, improvement in a systemic disease may reduce the cell death rate in lesions. Normalising the rate of cell death in lesions reduces the potential of cancer formation. In parallel, the same effect is provided in the case of cell senescence, where the oncogene-induced senescence will be halted [42].

Earlier treatment options for OLP and OL were limited and reduced to topical treatment. In this investigated population, OLP lesions improved within three to six months, whereas OL lesions improved less rapidly, over six months. In the course of the previously mentioned local and systemic treatments, however, leukoplakia changed their phenotype to homogeneous white lesions in each case, representing a lower risk for malignant transformation. Improvement was most impressive in OLP patients with previous systemic disorders that were either undiagnosed or not optimally controlled, subsequent to the introduction of appropriate therapy. Overall, it was found that the improvement in background systemic diseases has a positive effect on the regression of precancerous lesions.

As comorbid disease treatment was initiated for these patients in cooperation with physicians, other specialists, and home doctors, we could increase the prevalence of lesion elimination compared to previous studies. Oral medicine specialists repeatedly performed lesion check-ups.

The same process was revealed in the pathogenesis of diabetes and atherosclerosis, the latter belonging to cardiovascular diseases. In all the above-mentioned diseases, there is increased Th1 activity relative to Th2. On one hand, this stage of pathogenesis seems to be the common factor through which these diseases may be linked [43]. On the other hand, atherosclerosis is related to hyperlipidaemia, which, along with diabetes, is associated with metabolic syndrome. An association between erosive-type OLP, diabetes mellitus and arterial hypertension was first reported by Grinspan in 1996 [44]. Earlier studies hypothesised that the association of erosive-type OLP with diabetes and arterial hypertension could be attributable to the iatrogenic effects of the drugs used for diabetes and hypertension treatment [45]. In case of the atrophic–erosive OLP, an additional effect may exist, as these atrophic–erosive lesions exhibit increased rates of apoptosis and altered senescence. On the one hand, this is possibly because of increased rates of Th1 cell activity and on the other hand, because of increased tissue collagenase activity [31].

In the pathogenesis of autoimmune conditions, an overactivation of the Th2 cell population can occur. Hypothetically, this event can counteract or augment the Th1 overactivation, depending on the cytokine pattern. The Th1 and Th2 cells exist in close interaction [46]. Glucocorticoids do suppress the Th1 axis, and there is a shift to Th2-mediated immunity rather than a general immunosuppression. This is achieved by inhibiting the production of Il-12, INFα, INFγ, and TNFα. Practically, this is how acute and chronic stress can affect disease formation [47].

In the case of OL, the beneficial effect of comorbid disease improvement is mainly attributable to regaining regenerative abilities. The subepithelial lymphocyte infiltration, which in this case is mainly Th2, refers to the presence of mechanical, chemical irritations (effects of smoking), infections, and stress.

Conclusion: Local treatment alone does not yield satisfactory results. A systemic approach, or, as previously said, a ‘holistic’ approach, would provide better results.

## Figures and Tables

**Table 1:** Patient investigations.

1. Medical history	In assessing current complains, targeted questioning and a review of available medical documents were conducted
2. General physical examination	Inspection, palpation, auscultation, measurement of weight and height, pulse rate, and blood pressure (R/R)
3. Oral and dental examination	Inspection of mucous membranes Dental charting—registration of materials Taking mucosa samples with Cytobrush for HPV testing
4. Blood tests	CRP, ASO CBC, DBC, concentration of serum iron, transferring, ferritin, vitamin B12, and folate ‘Liver Function’ test: serum bilirubin level (direct + total), activities of ALT, AST, GGT, and LDH enzymes Antibodies against HBC and HCV were also examined Levels of T3, T4, and sTSH, as well as serum levels of anti-TPO, anti-TG cholesterol, LDL, HDL, and triglycerol, were measured Fasting blood glucose level, oral glucose tolerance test, HbA1c
5. Allergy test	Patch test for hypersensitivity reaction towards dental materials
6. Histology	HE stains of biopsy specimens and immunofluorescence labelling in case of bullous forms of OLP
7. Special examinations	Patients with erosive–atrophic lichen lesions and negative blood and allergy test results were referred to specialists to investigate hidden ailments, such as tumours and autoimmune diseases, involving specific diagnostic methods

Abbreviations: HPV = human papilloma virus, CRP = C-reactive protein, ASO = anti-streptolysin O, CBC = complete blood count, DBC = differential blood count, ALT = alanin-aminotransferase, AST = aspartate-aminotransferase, GGT = gamma-glutamyltransferase, LDH = lactate dehydrogenase, sTSH = sensitive thyroid stimulating hormone, anti-TPO = anti thyroidperoxidase antibody, anti-TG = anti thyroglobuline antibodiy, LDL = low density lipoprotein, HDL = high density lipoprotein, OLP = oral lichen planus.

**Table 2:** Prevalence of comorbid diseases in and tumours found throughout the course of this study were also registered.

	Control *n* = 139 (%)	OLP *n* = 144 (%)	OL *n* = 53 (%)	*p* Value	Total OLP + OL (*n* = 197)
non-erosive–erosive OLP; homogenous-non-homogenous OL	-	93/51	36/17		-
current smoker	7 (5)	10 (6.9)	5 (9.4)	N.S	15
cardiovascular diseases	57(41)	80 (55)	26 (49)	N.S	106
anaemia	24 (17.2)	49 (34)	16 (30)	N.S	55
iron deficiency	**15 (10.7)**	**54 (37.5)**	**19 (35.8)**	*p* = 0.00 (ANOVA)	63
vitamin B deficiency	8 (5.7)	8 (5.5)	6 (11.3)	N.S	14
diabetes	6 (4.3)	**33 (22.9)**	5 (9.4)	*p* = 0.01 (post hoc)	38
liver disorder	12 (8.6)	32 (22.2)	**16 (30.1)**	*p* = 0.02 (post hoc)	48
thyroiditis	0 (0)	12 (8.3)	4 (7.5)	N.S.	16
thyroid function	10 (7.1)	**38 (26.3)**	10 (18.8)	*p* = 0.05 (post hoc)	48
desquamative gingivitis	0 (0)	25 (17.3)	8 (15)	N.S.	33
lip involvement	0 (0)	8 (5.5)	2 (3.7)	N.S.	10
autoimmune disease	7 (5)	27 (18.7)	11 (20.7)	N.S.	38
allergy to dental materials	6 (4.3)	6 (4.1)	2 (3.7)	N.S.	8
HPV present in lesion cells (except controls: buccal mucosa and tongue)	**2 (1.4)**	**40 (27.7)**	**19 (35.8)**	0.00 (ANOVA)	59

Groups’ values with significant differences highlighted in bold. N.S. = non significant differences.

**Table 3:** Prevalence of comorbid diseases in OLP and OL subtypes.

	Non-Erosive–Non-Atrophic OLP (*n* = 93)	Erosive–Atrophic OLP (*n* = 51)	Homogenous OL (*n* = 36)	Non-Homogenous OL (*n* = 17)
current smoker	2 (2.1)	3 (5.8)	8 (22.2)	2 (11.7)
cardiovascular diseases	50 (53.7)	30 (58.8)	19 (52.7)	7 (41.1)
anaemia	34 (36.5)	15 (29.4)	10 (27.7)	6 (35.2)
iron deficiency	35 (37.6)	19 (37.2)	11 (30.5)	8 (47)
Vitamin B deficiency	5 (5.3)	3 (5.8)	3 (8.3)	3 (17.6)
diabetes	**14 (15)**	**19 (37.2)**	3 (8.3)	2 (11.7)
liver disorder	17 (18.2)	15 (29.4)	10 (27.7)	6 (35.2)
thyroiditis	6 (6.4)	6 (11.7)	2 (5.5)	2 (11.7)
thyroid function	26 (27.9)	12 (23.5)	7 (19.4)	3 (17.6)
desquamative gingivitis	14 (15)	11 (21.5)	3 (8.3)	5 (29.4)
lip involvement	3 (3.2)	5 (9.8)	2 (5.5)	0 (0)
autoimmune disease	17 (18.2)	10 (19)	6 (16.6)	5 (29.4)
allergy to dental materials	5 (5.3)	1 (1.9)	1 (2.7)	1 (5.8)
HPV present in lesion cells	24 (25.8)	18 (35.2)	12 (33.3)	7 (41.1)

Values of diabetes prevalence with significant differences (*p* = 0.004), highlighted in bold.

**Table 4:** OLP lesions: number and types of lesions at the beginning and by the end.

		Lesion Not Present by End Point	Non-Atrophic–Non-Erosive OLP Present by End Point	Atrophic–Erosive OLP by End Point	Total
	
non-erosive–non-atrophic OLP at the beginning		23	68	0	91
Erosive–atrophic OLP at the beginning		15	38	0	53
Total		38	106	0	144

**Table 5:** OL lesions: number and types of lesions at the beginning and by the end of the study period.

	Lesion Not Present by the End	Homogenous OL Present	Non-Homogenous OL by the End	Total
homogenous OL at the beginning	13	23	0	36
non-homogenous OL at the beginning	5	12	0	16
Total	18	35	0	53

## Data Availability

Data are contained within the article.
